# Supplementary material for: Integrated Analytical Tools for Accessing Acridones and Unrelated Phenylacrylamides from Swinglea glutinosa
Source: Molecules. 2019 Dec 30;25(1):153. doi: 10.3390/molecules25010153 (PMC6982866; doi:10.3390/molecules25010153)
Supplement: Supplementary file 1 [file molecules-25-00153-s001.pdf]

Supplementary Materials

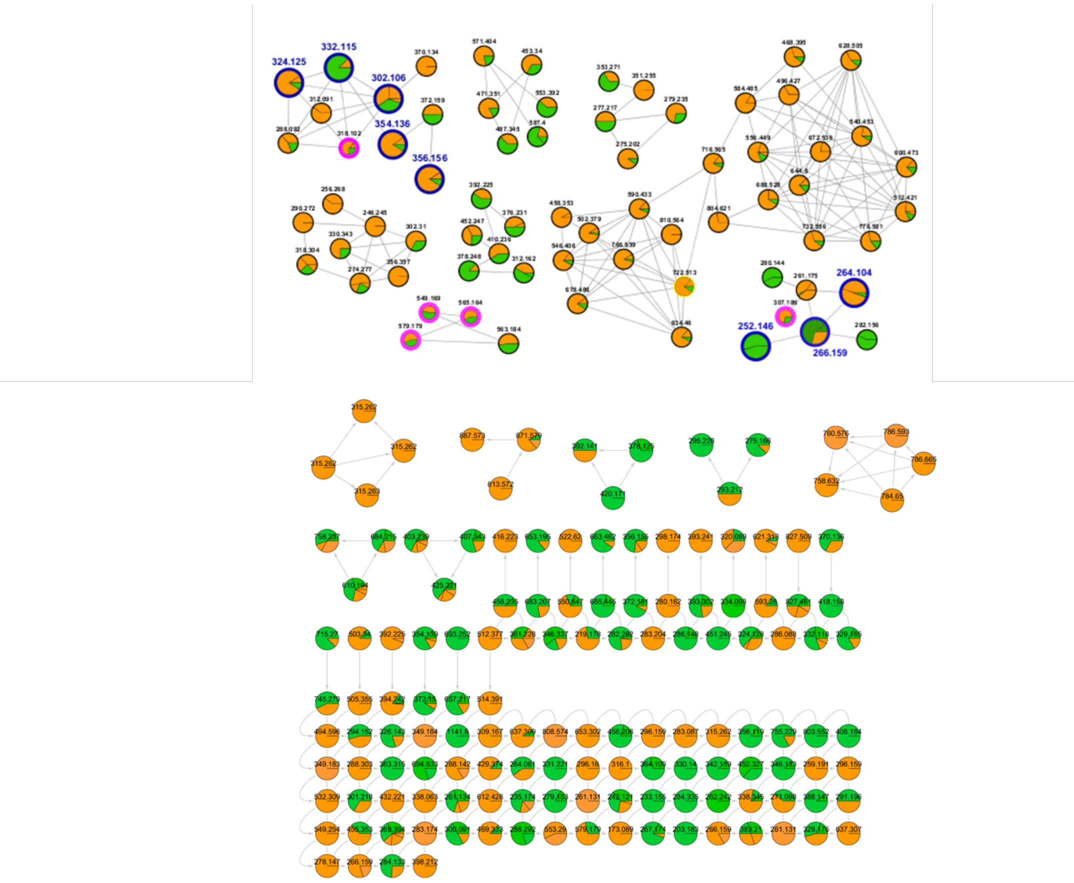

Figure S1: Molecular families obtained from *Swinglea glutinosa* extracts.

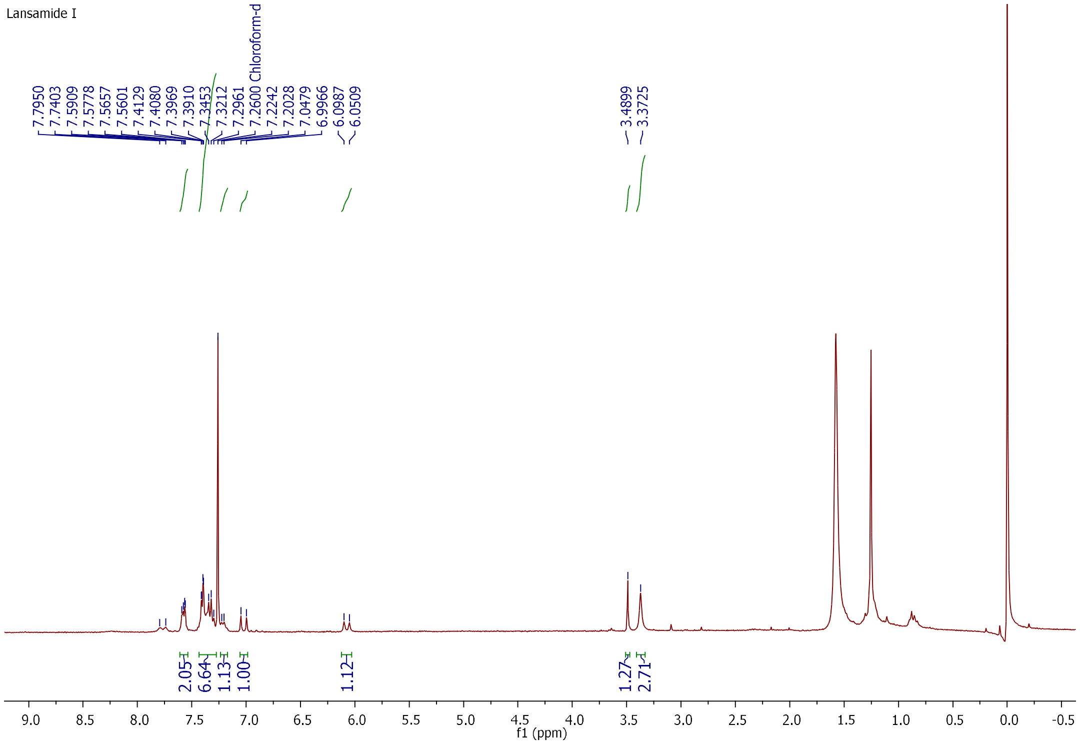

Figure S2: ^{1}H NMR spectrum (300 MHz, $CDCl_3$) of Lansamide I (**19**).

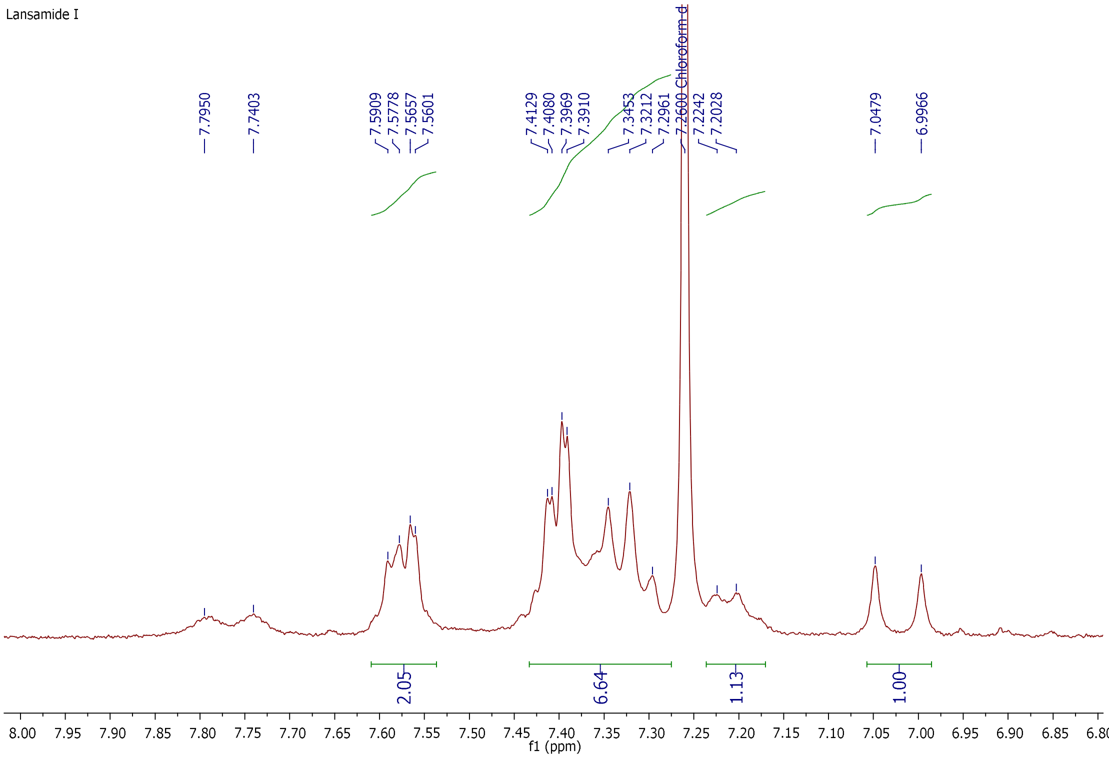

Figure S3: Enlarged ^{1}H NMR spectrum (300 MHz, $CDCl_3$) of Lansamide I (**19**).

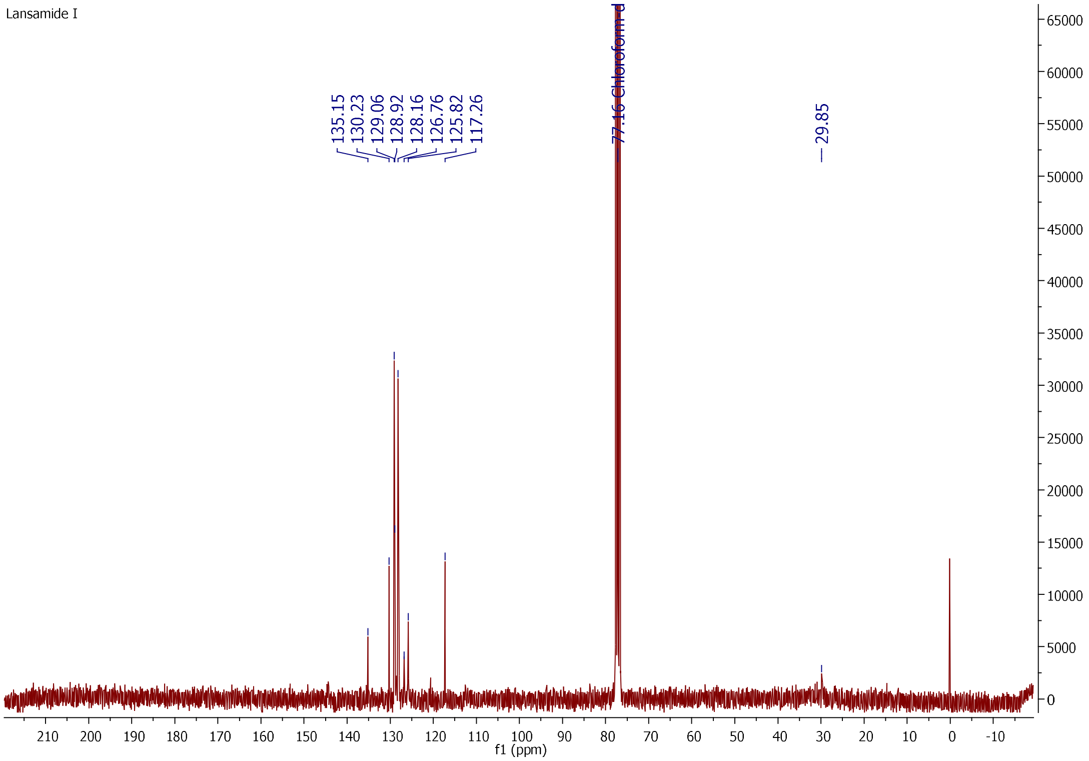

Figure S4: ^{13}C NMR spectrum (75 MHz, $CDCl_3$) of Lansamide I (**19**).

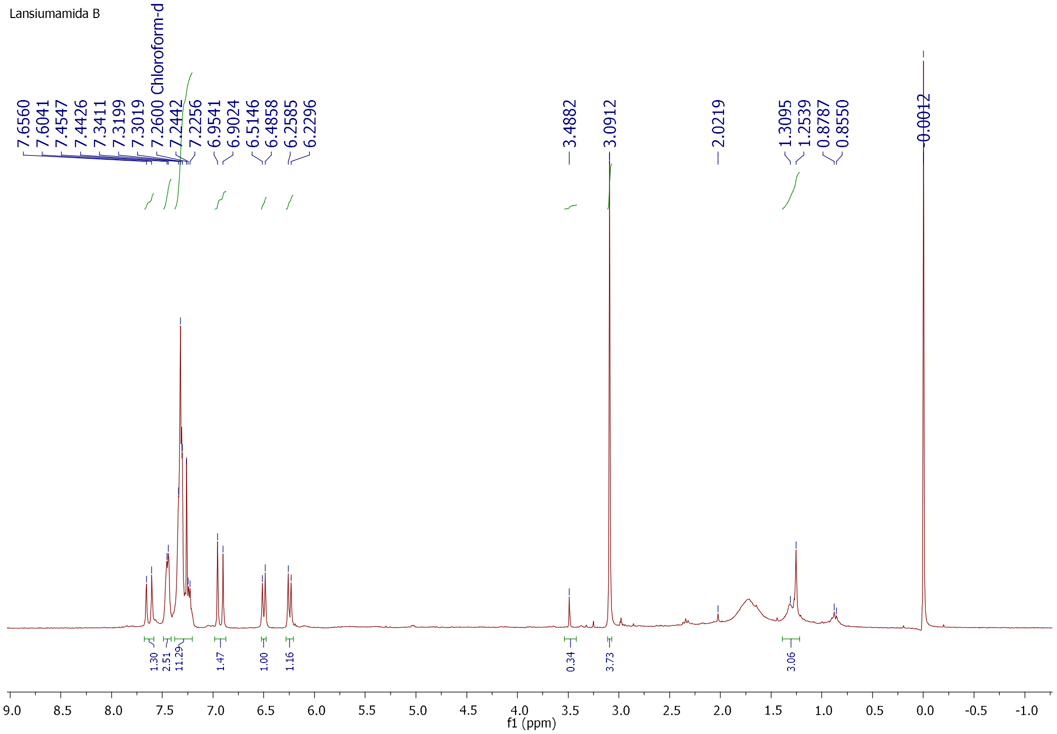

Figura S5: ^{1}H NMR spectrum (300 MHz, $CDCl_3$) of Lansiumamide B (**20**).

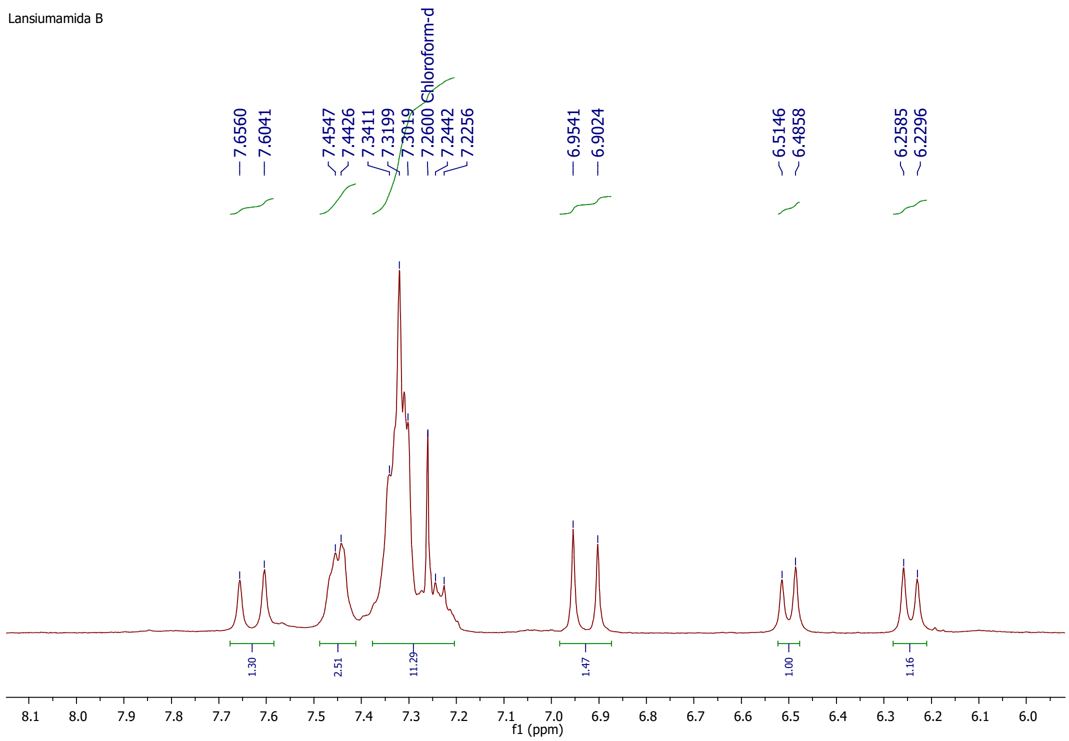

Figure S6: Enlarged ^{1}H NMR spectrum (300 MHz, $CDCl_3$) of Lansiumamide B (**20**)

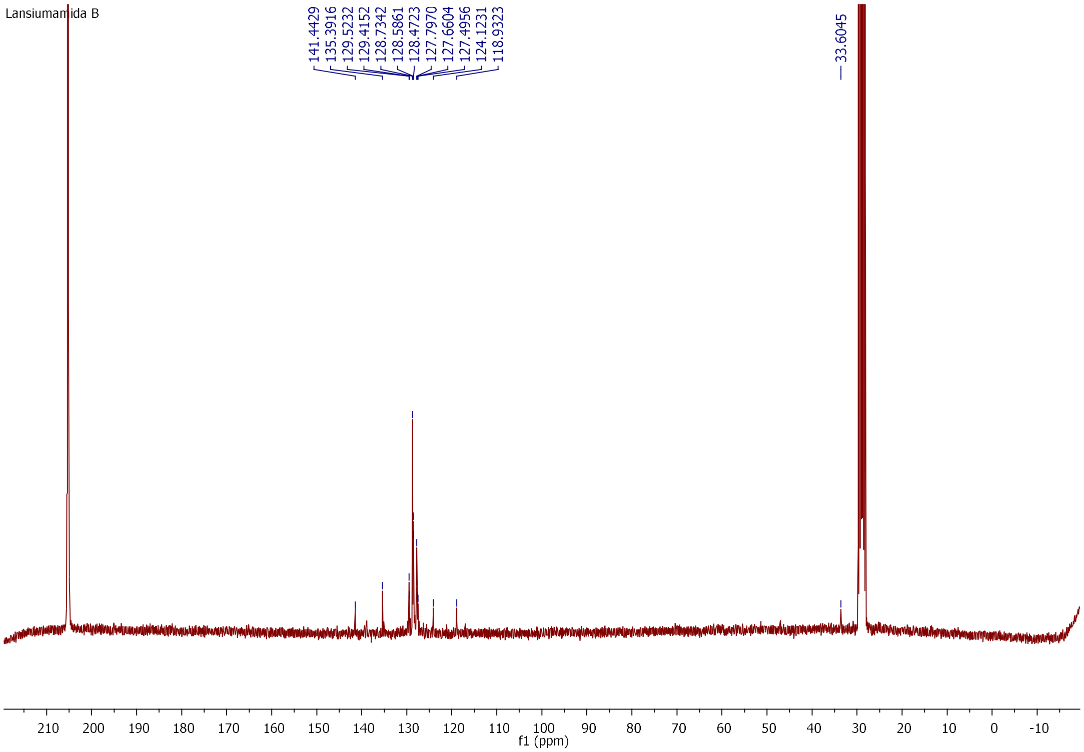

Figure S7. ^{13}C NMR spectrum (75 MHz, $CDCl_3$) of Lansiumamide B (**20**).

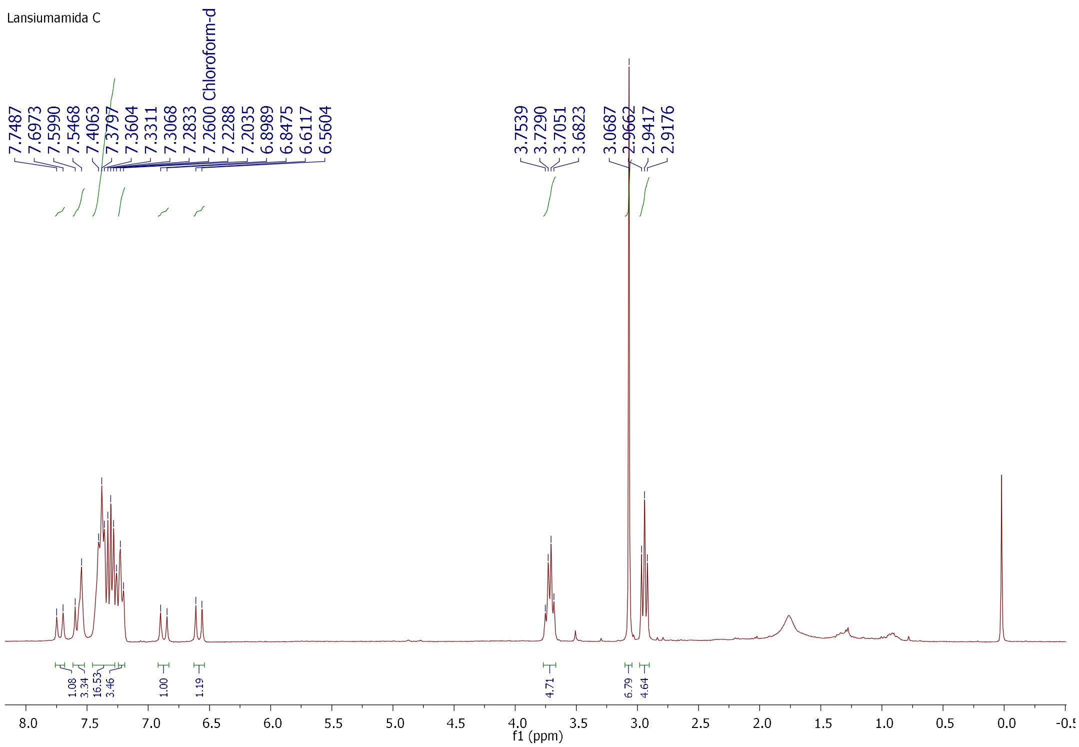

Figure S8. **^{1}H** NMR spectrum (300 MHz, $CDCl_3$) of Lansiumamide C (**21**).

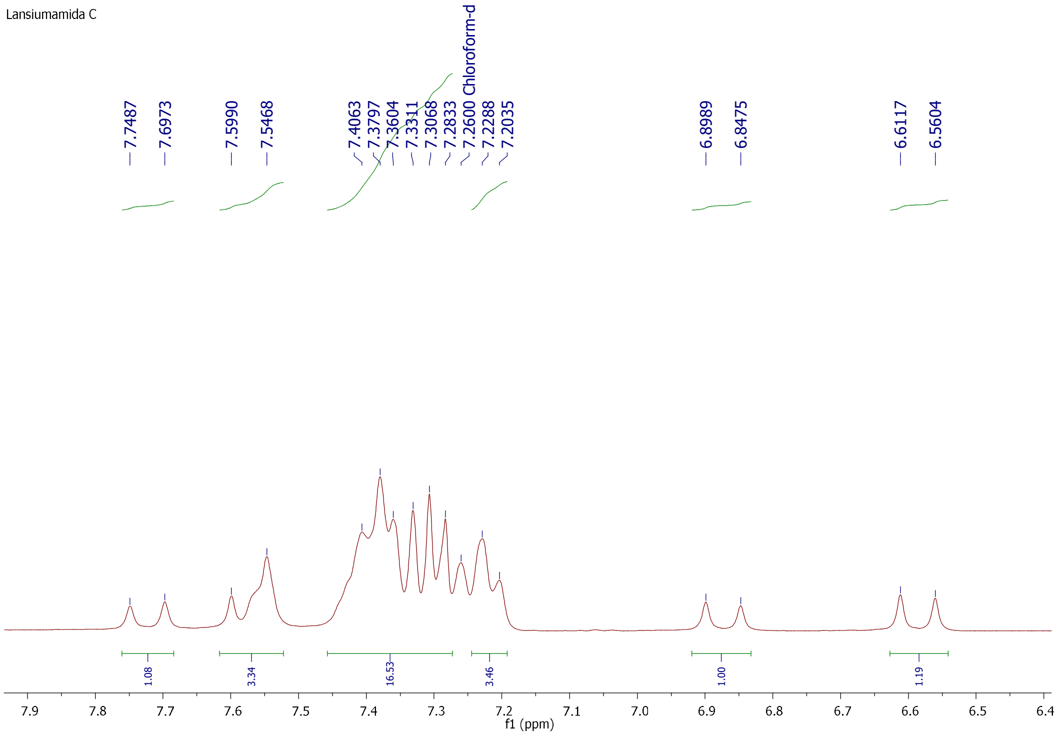

Figure S9. Enlarged ^{1}H NMR spectrum (300 MHz, $CDCl_3$) of Lansiumamide C (**21**).

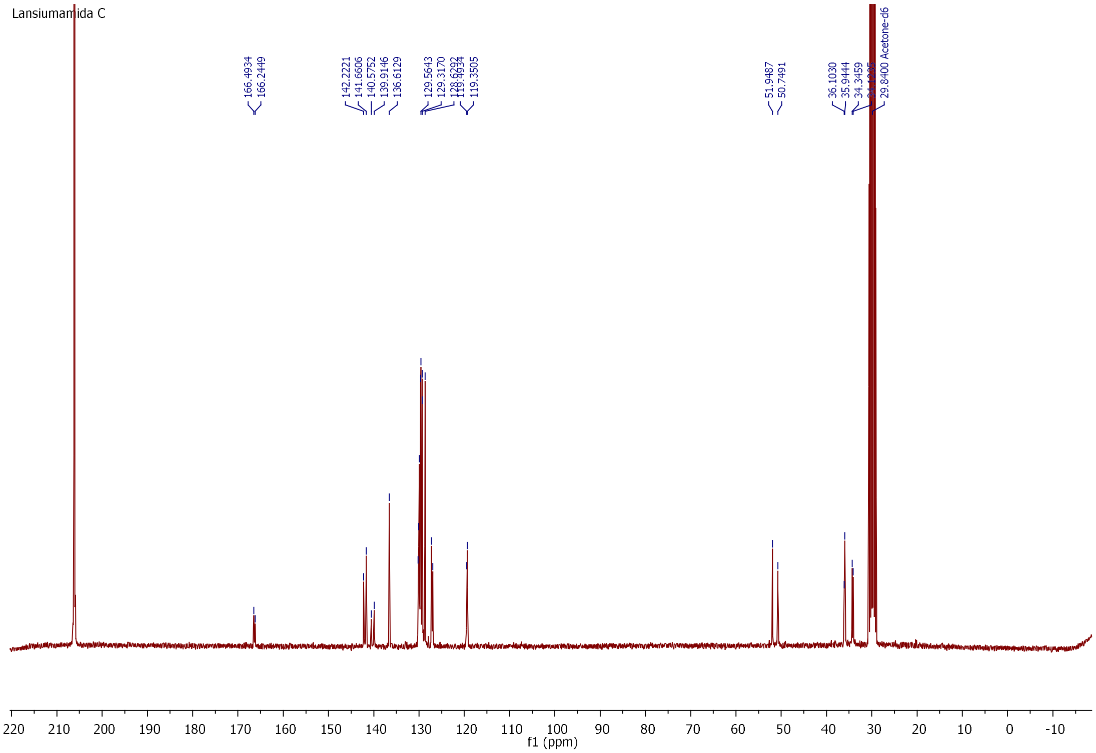

Figure S10. ^{13}C NMR spectrum (75 MHz, $CDCl_3$) of Lansiumamide C (**21**).

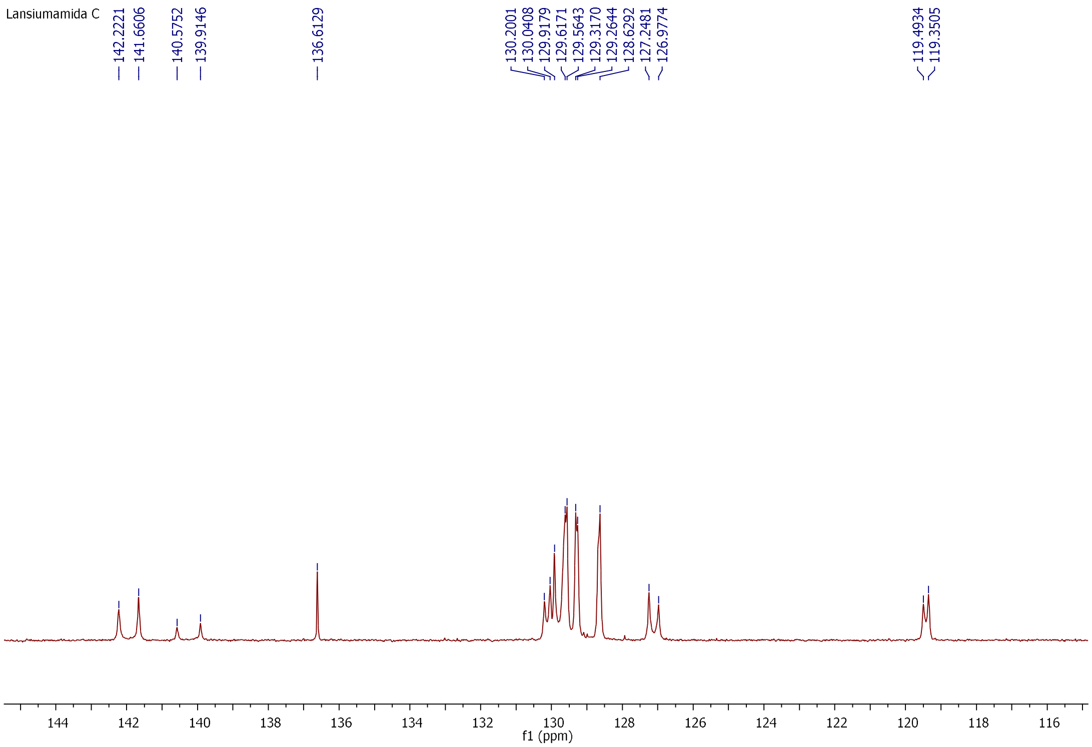

Figure S11. Enlarged ^{13}C NMR spectrum (75 MHz, $CDCl_3$) of Lansiumamide C (**21**).

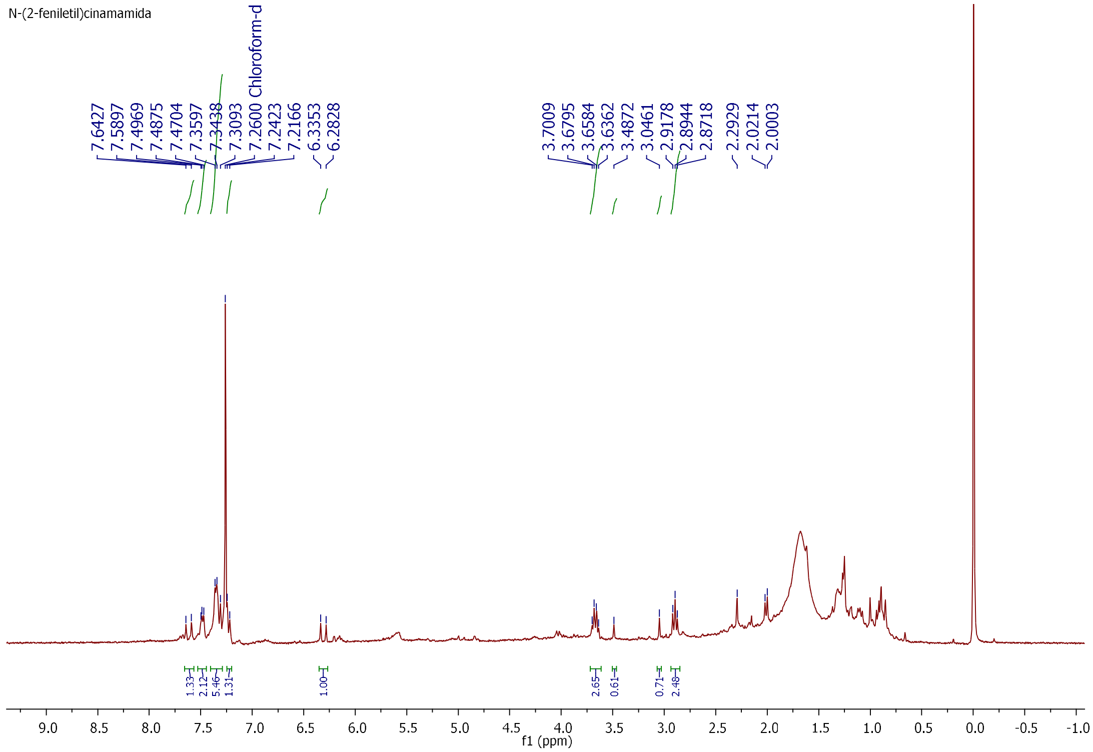

Figure S12. 1^{1}H NMR spectrum (300 MHz, $CDCl_3$) of *N*-(2-fenylethyl)-cinnamamide **(22).**

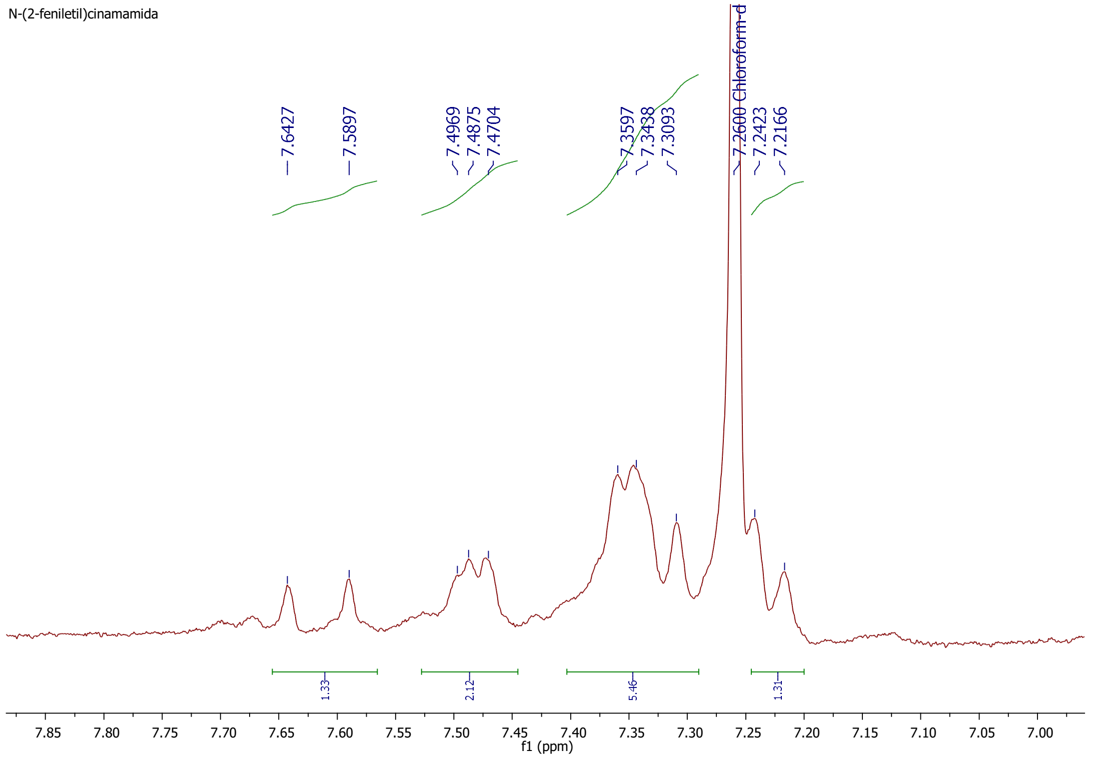

Figure S13. Enlarged ^{1}H NMR spectrum (300 MHz, $CDCl_3$) of *N*-(2-fenylethyl)-cinnamamide (**22**).

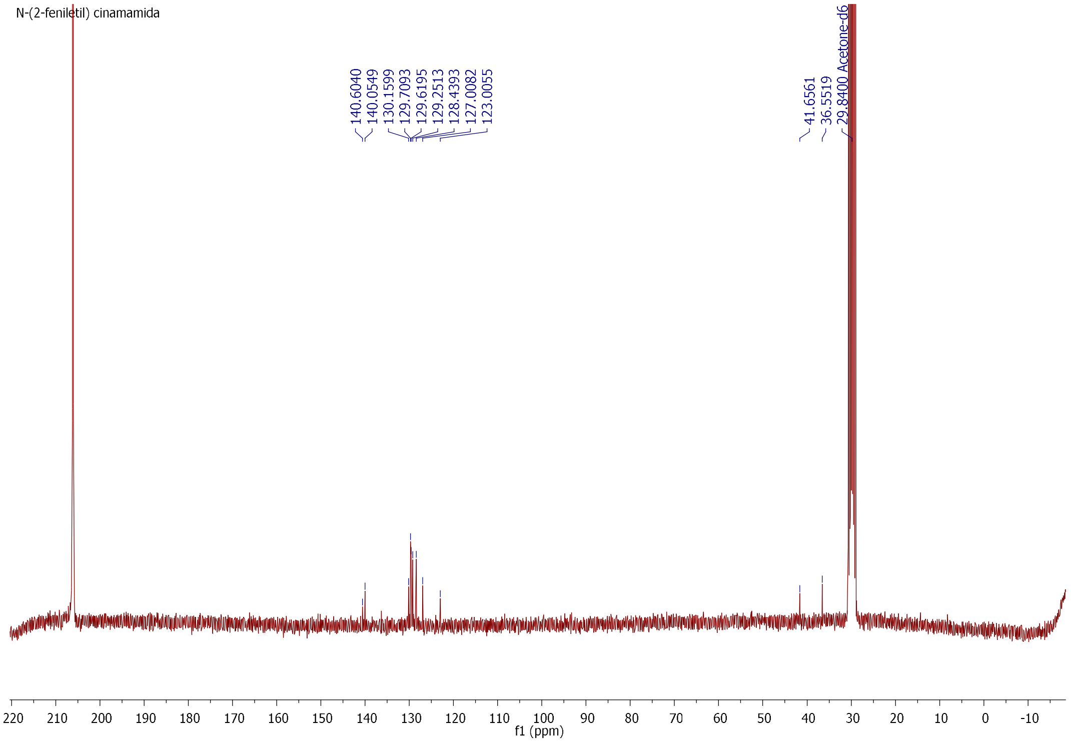

Figure S14. ^{13}C NMR spectrum (75 MHz, $CDCl_3$) of *N*-(2-fenylethyl)-cinnamamide (**22**).

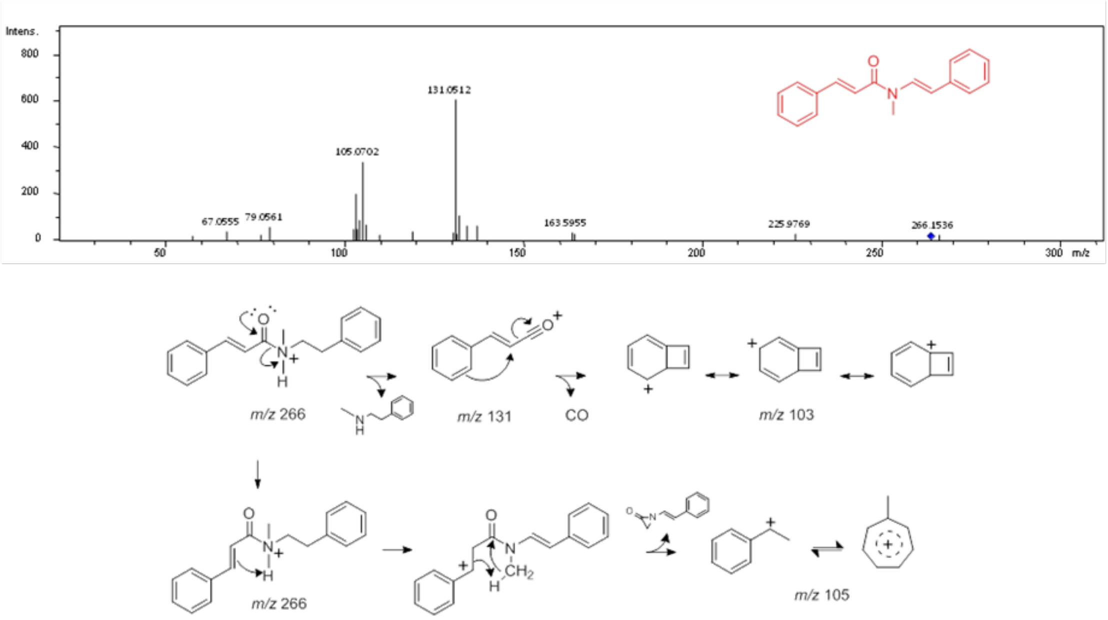

Figure S15. Fragmentation scheme for compound (**19**).

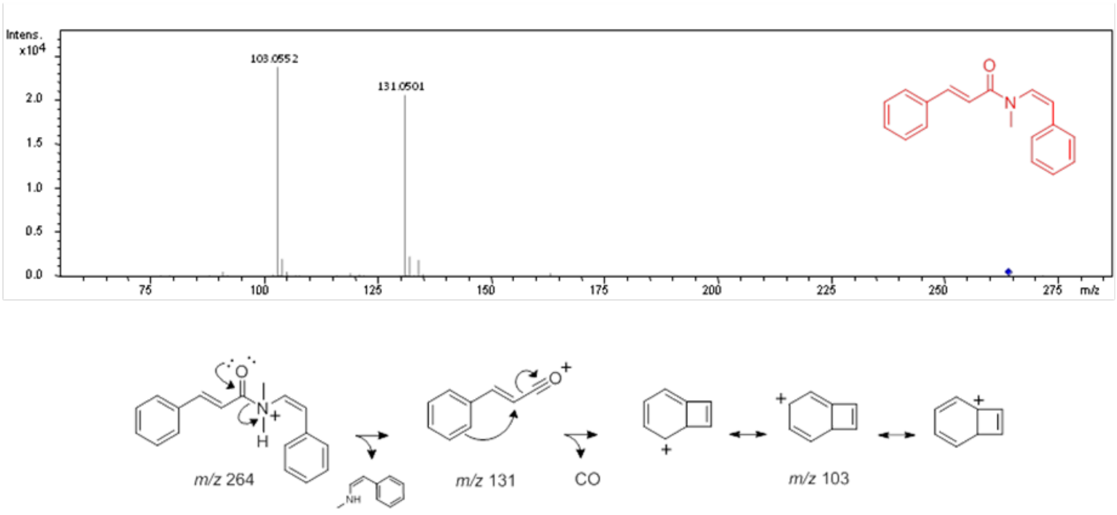

Figure S16. Fragmentation scheme for compound (**20**).

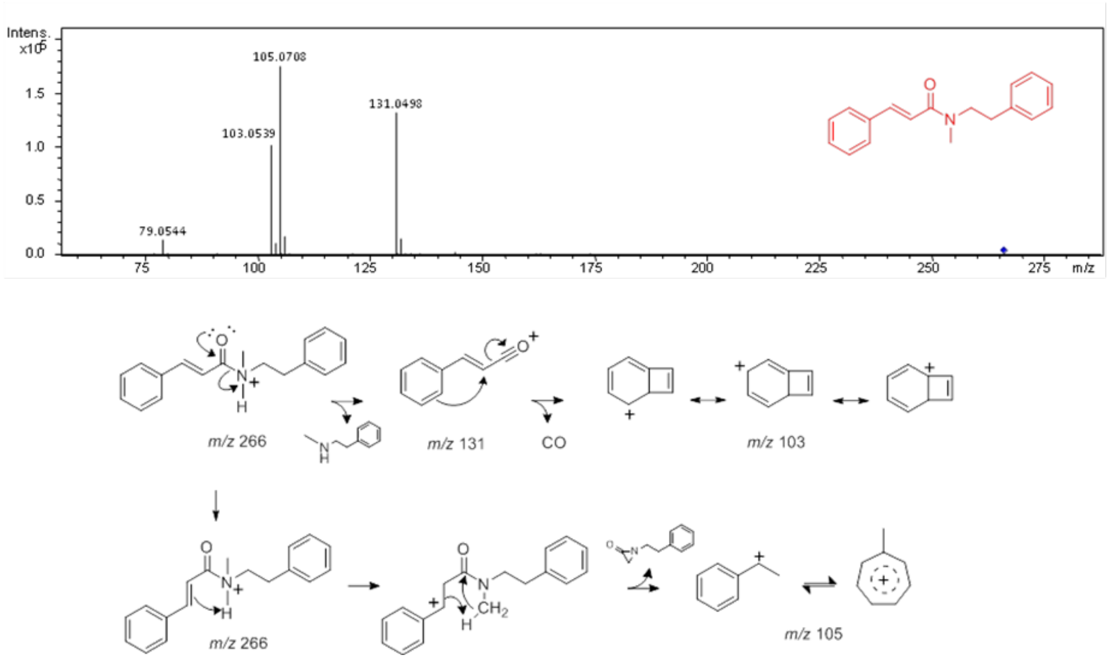

Figure S17. Fragmentation scheme for compound (**21**).

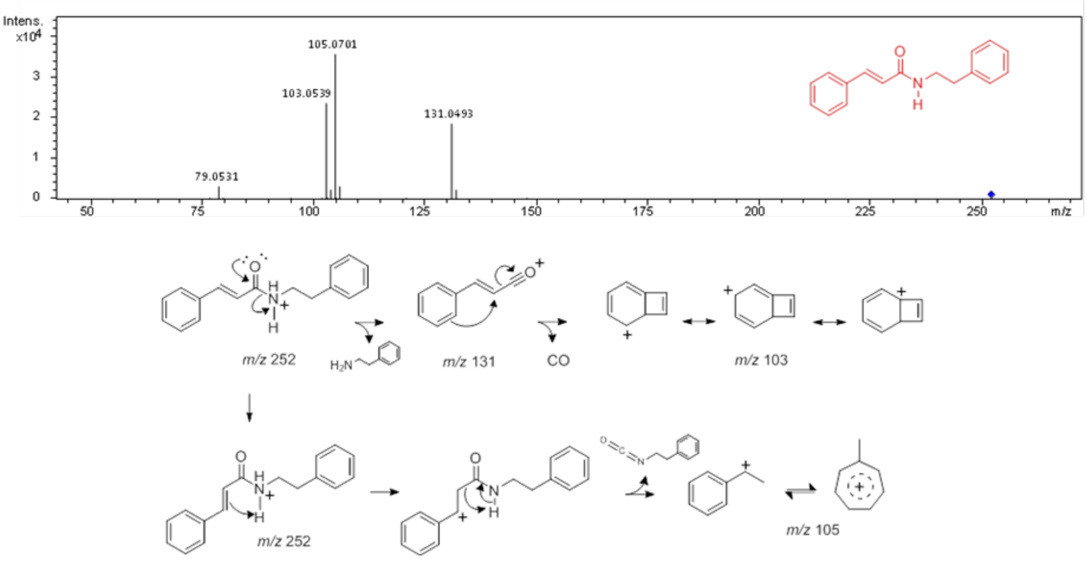

Figure A18. Fragmentation scheme for compound (**21**).
